# New opportunities for light-based tumor treatment with an “iron fist”

**DOI:** 10.1038/s41377-022-00762-3

**Published:** 2022-03-21

**Authors:** Riccardo Marin, Erving Ximendes, Daniel Jaque

**Affiliations:** Nanomaterials for Bioimaging Group (NanoBIG), Departamento de Física de Materiales, Facultad de Ciencias, Universidad Autónoma de Madrid, Madrid, 28030 Spain

**Keywords:** Applied optics, Optical physics

## Abstract

The efficacy of photodynamic treatments of tumors can be significantly improved by using a new generation of nanoparticles that take advantage of the unique properties of the tumor microenvironment.

The exploration of visible and near-infrared photons (henceforth, light) for diagnostic and therapeutic purposes is motivated by several considerations. For starter, the use of non-ionizing radiation has great appeal from a safety standpoint, reducing the need for protective measures or equipment on the patient and operator ends alike. Light sources and optical detectors are much more accessible and affordable than X-ray, magnetic resonance, and nuclear-medicine systems for imaging and therapy, both in terms of acquisition and maintenance. The real-time capabilities of light-based imaging approaches are another alluring aspect. The implementation of ever less invasive endoscopes and catheters^1^, then, alleviates the Achille’s heel of these techniques: the shallow penetration depth achievable—usually less than 1 cm.

Despite the appeal and demonstrated potential of light-based imaging and diagnostic approaches at the preclinical level, the vast majority of them are a far cry from the bedside. This bottleneck in terms of translation to the clinic is incredibly glaring in the case of nanotechnology-enabled approaches. The disproportion between the effort poured in the development of nanoparticle-based techniques and their use in humans is a matter that researchers, investors, and decision-makers should all look at with concern. Aside from worries regarding the intrinsic toxicity of nanoparticles and their fate within the body, one of the main obstacles that prevents their translation to the clinical practice is failure in demonstrating that their performance (in the case of imaging) and efficacy (in the case of therapy) is comparable to the one of conventional approaches. The burden is on us, the scientific community, to increase performance and efficacy, so to demonstrate the full potential of these methods. The recent work by Li and co-workers titled “*Light Amplified Oxidative Stress in Tumor Microenvironment by Carbonized Hemin Nanoparticles for Boosting Photodynamic Anticancer Therapy*” is a stride along this direction in the field of nanoparticle-assisted photodynamic tumor therapy^2^.

Photodynamic therapy (PDT) harnesses the capability of a species (photosensitizer) to generate, when photoexcited, reactive oxygen species (ROS) that induce localized damage to malignant cells^3^. Individually or in combination with conventional therapies like chemotherapy or radiotherapy, nanoparticle-assisted PDT was shown to lead to a significant reduction of tumor size. All this, of course, at the pre-clinical level. Nanoparticle-assisted PDT entails the intravenous or oral administration of nanoparticles. After accumulation in the tumor to be treated, through extravasation and/or active targeting, the nanoparticles are irradiated with laser light of adequate wavelength to generate ROS in situ and induce the death of tumor cells^4^. The therapy has minimal unwanted effects on surrounding healthy tissue, due to the localization of the nanoparticles and the possibility to accurately direct the excitation light. This elegant therapeutic approach sees its efficacy, however, crippled by several factors: (i) the number of nanoparticles that accumulate in the tumor is usually low due to a reduced targeting efficiency^5^, (ii) the properties of the tumor microenvironment limit the generation of ROS, and (iii) the optical properties of the tissues drastically reduce the amount of light that can reach the NPs housed intratumorally^6^. This is a “triple trouble” requiring innovative thinking to be fully overcome.

The recent publication by Li and co-workers^2^ introduces a significant advancement in the field of nanoparticle-assisted PDT by taking advantage of the synergy between several physicochemical effects (Fig. 1). The authors prepared phospholipid-encapsulated nanoparticles of carbonized hemin that under broadband excitation (400–700 nm) can generate ROS in the form of singlet oxygen (^1^O_2_) and hydroxyl radicals (•OH). The use of a broadband lamp is particularly relevant since it reduces the cost of the experimental setup required for the therapy. Because the tumor microenvironment is naturally poor in oxygen, a limit of PDT is that the reactions that leads to ROS generation usually goes to starvation rapidly. In the newly proposed system, the presence of Fe(III) in the nanoparticles—coming from the parent hemin—supports a Fenton-like redox reaction, which provides oxygen as the starting material for the generation of ^1^O_2_. Moreover, Fe(III) can react with and inactivate the antioxidant potential of glutathione. These combined effects, both originating from iron, offer a boost to this PDT approach, overcoming two major hurdles naturally imposed by the tumor microenvironment.

**Fig. 1:**
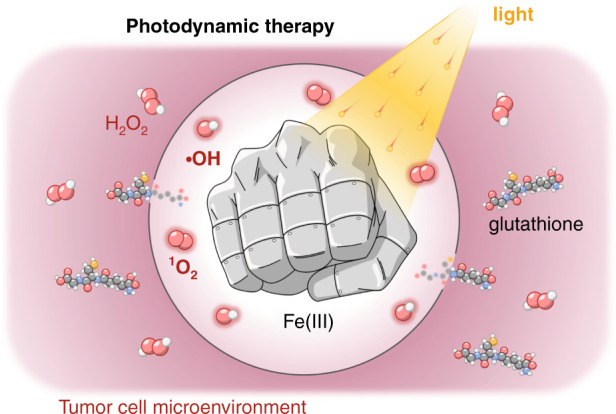
An “iron fist” to boost the performance of PDT. A pictorial representation of the action of Fe(III) in carbonized-hemin nanoparticles during PDT in terms of supporting ROS generation from H_2_O_2_ and glutathione depletion

The good performance of the approach during in vitro tests on cell lines is only a stepping stone towards the results obtained in vivo, which are especially impressive. Firstly, an excellent tumor targeting efficiency, when compared to the state of the art^7^, was observed by monitoring the nanoparticles’ fluorescence in organs ex vivo. Moreover, irradiation of the nanoparticle-targeted tumor with low light intensities (0.1 W/cm^2^) resulted in an almost 100% tumor inhibition rate.

Overall, Li and co-workers’ study constitutes a new, holistic way of understanding light-based tumor treatments, harnessing a synergy between the chemical properties of the tumor microenvironment and the optical properties of the nanoparticles^2^.

Future research directions in this field might and should include an attentive look at the clearance of the nanoparticles, since accumulation is a matter of concern in nanomedicine^8^. Yet, the benign composition of the nanoparticles (C and Fe) makes this a less critical point. Active targeting with tailored moieties could also be explored, aiming at an increased bioavailability of the nanoparticles and hence a more efficient PDT approach. Strategies to push the light absorption towards the near-infrared, where the photon–tissue interaction is reduced, could help reaching deeper within tissues^9,10^. Furthermore, the evaluation of the therapy efficiency as a function of irradiation wavelength could identify optimal wavelengths resulting from the interplay between nanoparticle absorption and wavelength dependence of ROS generation. The identification of such optimal wavelengths would enable the use of reduced irradiation doses and cost-effective monochromatic sources (such as LEDs)^11^. A combination of the studies above would cover all the critical angles and make nanoparticle-assisted PDT an even more attractive therapeutic approach, thus possibly sparking the interest of forward-looking physicians.
